# In vitro α-glucosidase inhibitory activity of *Tamarix nilotica* shoot extracts and fractions

**DOI:** 10.1371/journal.pone.0264969

**Published:** 2022-03-14

**Authors:** Mariane Daou, Nancy A. Elnaker, Michael A. Ochsenkühn, Shady A. Amin, Ahmed F. Yousef, Lina F. Yousef

**Affiliations:** 1 Department of Biology, Khalifa University, Abu Dhabi, United Arab Emirates; 2 Department of Chemistry, Khalifa University, Abu Dhabi, United Arab Emirates; 3 Biology Program, New York University in Abu Dhabi, Abu Dhabi, United Arab Emirates; 4 Center for Membranes and Advances Water Technology (CMAT), Khalifa University, Abu Dhabi, United Arab Emirates; Bangabandhu Sheikh Mujibur Rahman Agricultural University, BANGLADESH

## Abstract

α-glucosidase inhibitors represent an important class of type 2 antidiabetic drugs and they act by lowering postprandial hyperglycemia. Today, only three synthetic inhibitors exist on the market, and there is a need for novel, natural and more efficient molecules exhibiting this activity. In this study, we investigated the ability of *Tamarix nilotica* ethanolic and aqueous shoot extracts, as well as methanolic fractions prepared from aqueous crude extracts to inhibit α-glucosidase. Both, 50% ethanol and aqueous extracts inhibited α-glucosidase in a concentration-dependent manner, with IC_50_ values of 12.5 μg/mL and 24.8 μg/mL, respectively. Importantly, α-glucosidase inhibitory activity observed in the *T*. *nilotica* crude extracts was considerably higher than pure acarbose (IC_50_ = 151.1 μg/mL), the most highly prescribed α-glucosidase inhibitor on the market. When *T*. *nilotica* crude extracts were fractionated using methanol, enhanced α-glucosidase inhibitory activity was observed in general, with the highest observed α-glucosidase inhibitory activity in the 30% methanol fraction (IC_50_ = 5.21 μg/mL). Kinetic studies further revealed a competitive reversible mechanism of inhibition by the plant extract. The phytochemical profiles of 50% ethanol extracts, aqueous extracts, and the methanolic fractions were investigated and compared using a metabolomics approach. Statistical analysis revealed significant differences in the contents of the crude extracts and fractions and potentially identified the molecules that were most responsible for these observed variations. Higher α-glucosidase inhibitory activity was associated with an enrichment of terpenoids, fatty acids, and flavonoids. Among the identified molecules, active compounds with known α-glucosidase inhibitory activity were detected, including unsaturated fatty acids, triterpenoids, and flavonoid glycosides. These results put forward *T*. *nilotica* as a therapeutic plant for type 2 diabetes and a source of α-glucosidase inhibitors.

## Introduction

Type 2 diabetes accounts for 90% of the total diabetes cases and the overall rise in prevalence over the years [1]. It is characterized by insulin resistance and beta-cells dysfunction leading to reduced glucose uptake and postprandial hyperglycemia [2]. Increased numbers of type 2 diabetes cases particularly among younger adults were found to be triggered by reduced physical activity and the overall rise in the prevalence of obesity [1, 3]. Carbohydrate-rich diets and frequent consumption of high glycemic index foods were found to be associated with increased glucose and insulin levels (12% and 9%, respectively) and a higher risk of type 2 diabetes [4, 5]. In contrast, low-carbohydrate ketogenic diets, showed marked improvement in glycated hemoglobin, fasting glucose, fasting insulin, and body weight, leading to the discontinuation or reduction of diabetes medications in 95.2% of the participants [6].

From a public health perspective, preventing diabetes is the ultimate aim. However, the exact mechanisms linking type 2 diabetes to obesity and diet are not entirely understood, and the disease is sometimes triggered by genetic predispositions, making healthy diet and exercise alone inefficient at preventing the disease [7]. Failing to maintain normal blood glucose levels is responsible for the chronic and fatal complications of diabetes including cardiovascular disease, diabetic retinopathy, nephropathy, and neuropathy [8–11]. To reduce the damage caused by diabetes, there is a need for medications that can help patients regulate blood sugar levels. All approved diabetes medications on the market aim to normalize blood glucose levels by targeting the different mechanisms and enzymes involved in glucose homeostasis. One particularly interesting class of diabetic drugs is α-glucosidase inhibitors. These drugs decrease postprandial hyperglycemia by reversibly inhibiting α-glucosidase enzymes in the brush border of the small intestine responsible for the breakdown of complex carbohydrates to glucose [12]. Unlike drugs that regulate blood glucose more strictly (insulin, sulfonylureas, and meglitinides), α-glucosidase inhibitors are not associated with weight gain and hypoglycemia [13]. This class of drugs was also found to reduce the risk of type 2 diabetes by 35.6%, and this effect was independent of age, sex, and body mass index, making α-glucosidase inhibitors effective across a wide spectrum of patients [14]. α-glucosidase inhibitors are further believed to impart a vasoprotective effect by reducing postprandial glucose level, which is correlated with endothelial dysfunction, cardiovascular disease, and stroke [15, 16].

Although α-glucosidase inhibitors have been in clinical use since 1990, only three are available on the market: acarbose, miglitol, and voglibose [17–19]. They are frequently associated with gastrointestinal side effects, mainly flatulence and diarrhea, which is a common cause for the discontinuation of the treatment among diabetic patients [14]. In addition, several case reports described drug-drug interactions of acarbose particularly with digoxin (reduced absorption) and warfarin (increased absorption), which are used in the treatment of heart conditions and as an anticoagulant, respectively [20, 21]. When long-term medications in the case of chronic diseases such as type 2 diabetes are required, treatment efficacy, convenience, accessibility, and the safety of the therapy are equally considered. Exploring new effective and safe therapeutic agents for the prevention and treatment of diabetes is therefore important.

Traditional herbal formulations are generally considered a good alternative as they are less toxic, not associated with serious side effects, and can be produced at a lower cost. The antidiabetic effect, and particularly the α-glucosidase inhibitory activity of different plants and plant-derived metabolites has been widely studied and reviewed [22, 23], with some formulations already prescribed by physicians and available on the market [24, 25]. Active phytochemicals acting as α-glucosidase inhibitors include alkaloids, phenolic acids, flavonoids, terpenoids, anthocyanins, and their glycosides [26, 27]. The extraction method applied plays a very important role in determining the extraction efficiency and the obtained biological activities. The total phenolic content and antioxidant activity of *Tamarix aphylla* for example have been directly affected by the extraction solvent used [28]. The optimization of the extraction technique is also important. Microwave-assisted extraction of *T*. *chinensis* for instance has been optimized to obtain high extraction efficiency of flavonoids and increased antioxidant activity within a shorter extraction time compared to conventional methods [29].

*Tamarix spp*. has been traditionally used for the treatment of diabetes mellitus and recent reports have demonstrated that *T*. *aphylla* and *T*. *gallica* extracts exhibit a potent inhibitory effect on α-glucosidase [30, 31]. The compounds responsible for this activity were isolated from *T*. *gallica* ethanol extracts and included quercetin, rhamnetin, rhamnazin, tamarixetin, and kaempferol, and their O-methylated and glucuronosylated derivatives [31]. Aqueous leaf extracts of *T*. *nilotica* have previously shown a significant antihyperglycemic effect in diabetic rats [32], however, the ability of this plant to inhibit α-glucosidase has never been investigated. To identify new compounds that may be suitable for development as α-glucosidase inhibitors, we screened *T*. *nilotica* extracts for this activity. The *in vitro* inhibitory activities of aqueous and ethanol shoot extracts (TN extracts) were investigated and compared to the commonly prescribed α-glucosidase inhibiting drug acarbose. The kinetic parameters and type of inhibition were also determined. Aqueous crude extracts were fractionated in a 10–80% methanol gradient using solid-phase extraction, and the α-glucosidase inhibitory activities of the fractions were assayed. The phytochemical content of the extracts and fractions was investigated using a non-targeted metabolomics approach and the metabolites potentially responsible for the inhibition were identified. The results presented here provide compelling evidence that *T*. *nilotica* is a source of α-glucosidase inhibitors that could potentially be used to treat type 2 diabetes.

## Materials and methods

### Reagents and plant extracts

α-glucosidase from *Saccharomyces cerevisiae*, 4-nitrophenyl α-D-glucopyranoside (pNPG), p-nitrophenyl (pNP), and acarbose were purchased from Sigma-Aldrich (St. Louis, Missouri, USA). Fresh shoots of the plant were collected in Abu Dhabi, UAE, air-dried at 60 ˚C for 24 h, and ground into a fine powder using a coffee grinder (Moulinex AR110O27) before storage at 22 ˚C in airtight plastic bags [33]. Powdered biomass (3 g) was mixed with distilled water or 50% ethanol (100 mL) and the suspension (in a sealed container) was exposed to microwaves (AFMW205M, Aftron) for 3 cycles of 30, 10, and 5 s (231 W). Between each cycle, the suspension was manually shaken and allowed to cool down to room temperature before proceeding with the next cycle. Throughout this process, the temperature of the sample did not exceed 60°C. Extraction yields were determined by filtering the extracts on pre-weighted Whatmann GF/C filter paper (GE Life Sciences, Dubai, UAE) and drying at 60°C for 48 h. Filtered water (TNW) and 50% ethanol (TN50E) extracts were dried in a rotary evaporator (IKA RV 10 digital V, IKA-Werke GmbH & Co, Staufen, Germany) at 40°C, lyophilized at -100°C and 0.250 Pa for 48 h (Labconco, MO, USA) and stored at -20°C until use. All extractions were carried out in triplicates.

Lyophilized TNW extract was further fractionated by solid-phase extraction using C-18 SPE cartridges (WAT36905 6CC 1 g, Waters, MA, USA) pre-conditioned with methanol. The loaded extract was sequentially eluted with increasing concentrations of methanol in water (10% to 80% methanol with 10% increment). The flow-through was eluted with 100% methanol (TNW100M). Collected fractions (designated as TNW10M to TNW80M) were lyophilized as described above in pre-weighted tubes and the yields were determined. Fractionation was carried out in triplicate.

The lyophilized extracts (TNW and TN50E) and fractions (TNW10M to TNW80M) were dissolved in 100 mM sodium phosphate buffer pH 6.9 to prepare stock solutions at 2.5 mg/mL. The solutions were stored at -20°C.

### Total phenolic content

The total phenolic content assay was carried out using the Folin-Ciocalteu reagent as previously described with some modifications [34]. Gallic acid was used as a standard, and the standard curve (from 0.01 to 0.1 mg/mL diluted in methanol) was generated at 765 nm. The sample extract (0.5 mg) was added to 2.5 mL 10% Folin-Ciocalteu reagent and 2 mL of 7.5% Na_2_CO_3_ solutions vortexed and incubated in the dark for 1 h at room temperature before absorbance measurement at 765 nm. Results were expressed as milligrams of gallic acid equivalent (GAE) per g dry extract (mg GAE/g dry extract). The experiments were performed on *T*. *nilotica* crude extracts and fractions in triplicate.

### Antioxidant activity

The 1,1, Diphenyl-2-Picrylhydrazyl (DPPH) assay was performed to measure the antioxidant capacity of the extracts and fractions [35]. DPPH assay was carried out using a dilution series of plant extract (0.6–0.02 mg/mL). A volume of 0.5 mL from each concentration was mixed with 0.3 mL of 0.5 mM DPPH and 3.5 mL of methanol. Ascorbic acid was used as a reference standard and dissolved in distilled water to make the stock solution with the same concentration as the extract. The control sample contained the same volume without the extract. The blank contained 95% methanol. The reactions were incubated in the dark at room temperature for 30 min before absorbance measurement at 517 nm. The resulting IC_50_ value, which is the concentration of extract that scavenged 50% of the DPPH radical was determined. The % DPPH free radical scavenging was calculated as follow:

$$DPPH\;scavenging\;effects\;(\%)=\frac{{Absorbance}_{control}-{Absorbance}_{reaction}}{{Absorbance}_{control}}\times 100$$

### α-glucosidase inhibition assay

The inhibition of α-glucosidase enzyme was determined in 100 mM sodium phosphate buffer pH 6.8 by reacting 0.1 U/mL of the enzyme with 1.25 mM pNPG in the absence or presence of TN extracts (10 μg/mL) in a final reaction volume of 200 μL at 37°C. The reaction in the presence of acarbose (10 μg/mL) was used as a positive control. One unit of α-glucosidase activity is defined as the amount of α-glucosidase required to liberate one μmole of pNP from pNPG per minute. The reaction was initiated by adding pNPG and the released pNP was determined by measuring absorbance at 410 nm using a spectrophotometer (Fluostar Optima Microplate Reader, BMG Labtech, Offenburg, Germany) after 10 min of reaction time. Background readings were eliminated by subtracting the absorbance of the mixture without enzyme. The measured absorbance was considered directly proportional to the enzymatic activity and α-glucosidase inhibition was determined as follow:

$$Enzyme\;inhibition\;(\%)=\left(\frac{{Absorbance}_{negative\;control}-{Absorbance}_{reaction}}{{Absorbance}_{negative\;control}}\right)\times 100$$

The inhibition assay (with varying concentrations of inhibitor) was also used to determine the concentrations of TN extracts resulting in 50% inhibition (IC_50_ values) compared to acarbose. All measurements were carried out in triplicates.

### Kinetics of α-glucosidase inhibition and effect of incubation

α-glucosidase kinetic parameters were determined in the standard assay by increasing the concentration of pNPG (0–3 mM) in the absence and presence of acarbose or TN extracts (TNW and TN50E) at IC_50_. The reaction was followed spectrophotometrically at 410 nm for 10 min. The molar extinction coefficient of pNP was determined from the standard curve (4.38 mM^− 1^ cm^-1^) and was used to calculate the activity of the enzyme. The inhibition type was determined with Lineweaver-Burk plots and the kinetic parameters were obtained by nonlinear fitting to the Michaelis–Menten plot (Origin 2020b).

The effect of pre-incubation on the inhibitory activity of TN50E extract was evaluated by incubating the enzyme with different concentrations of extract in the reaction buffer. After 1 h of incubation at 37°C, the reaction was initiated, and α-glucosidase inhibition was determined as described above. The IC_50_ shift was calculated as follow: ${IC}_{50}\;shift=\frac{{IC}_{50}\;without\;incubation}{{IC}_{50\;after\;1\;hour\;of\;incubation}}$.

The reversibility of TN50E extract inhibition was determined by rapid-dilution assay. α-glucosidase at a concentration 100-folds higher than the one used in the standard assay was mixed with TN50E extract at a concentration 10-folds higher than its IC_50_. The mixture was incubated at 37°C for 30 min then diluted 100-folds into the standard reaction. The release of pNP over time was followed as described above and compared to the control without inhibitor. All measurements were carried out in triplicate.

### Thermal and pH stabilities of TN extracts

The pH stability of TNW and TN50E extracts was assessed by dissolving the extract in 100 mM citrate-phosphate buffer at different pH (pH 3, 7.8, and 8) and incubating for 4 h at 37°C before performing the standard α-glucosidase inhibition assay using IC_50_ equivalent of the inhibitor.

To determine thermal stability, TNW and TN50E extracts were incubated for 4 h at 25°C, 37°C, or 50°C before performing the standard α-glucosidase inhibition assay using IC_50_ equivalent of the inhibitor.

In both experiments, α-glucosidase inhibition was determined and compared to the control reaction before incubation. All measurements were carried out in triplicates.

### Metabolite analysis of TN extracts

Ultra-High-Performance Liquid Chromatography (UHPLC, Agilent 1290) coupled to quadrupole time-of-flight mass spectrometry (Q-ToF-MS, Bruker, Germany) was used to analyze the metabolite content of the TNW, TN50E, and TNW10M to TNW100M extracts as previously described [36]. Metabolites were separated on reversed-phase Eclipse Plus C18 column (50 mm × 2.1 mm ID) (Agilent, CA, US) with buffer A (MilliQ-H_2_O + 0.2% formic acid) and buffer B (acetonitrile + 0.2% formic acid) as mobile phases at a flow rate of 0.4 mL/min. Each elution cycle was a gradient starting at 90% buffer A and 5% buffer B and increasing over 18 min to 100% buffer B and holding at 100% buffer B for 2 min. All measurements were carried out in triplicates.

Eluted compounds were detected in the positive and negative ionization modes with the following parameters: mass range = 50–1300 m/z measured at 6 Hz; ESI source parameters: dry gas temperature = 220°C, dry gas flow = 10.0 L/min, nebulizer pressure = 2.2 bar, capillary voltage = 3000 V, end plate offset = 500 V; MS-ToF tuning parameters: funnel 1 RF = 150 Vpp, funnel 2 RF = 200 Vpp, isCID Energy = 0 eV, Hexapole RF = 50 Vpp, ion energy = 4.0 eV, low mass = 90 m/z, collision energy = 7.0 eV, pre-pulse storage = 5 μs. Data was processed using Metaboscape 3.0 (Bruker, Bremen, Germany).

Mass data acquisition was conducted with the T-Rex3D algorithm with an intensity threshold of 500 intensity counts and a minimum peak length of 10 spectra. Spectra were lockmass calibrated and features were only created if detected in a minimum of 3 samples. Molecular feature identification was based on LC-MS accurate mass and targeted MS^2^ fragmentation analysis of annotated features using an external library of 2000 metabolites (Bruker) along with an in-house library of over 100 plant metabolites. Positive and negative data were separately processed with the above parameters, merged afterward into one dataset, and exported as CSV (Comma delimited) (*.csv) file.

### Statistical analysis

The statistical analysis was performed using Metaboanalyst 5.0 software [37]. A t-test was carried out to examine metabolic differences between two samples. Principal component analysis (PCA) was used to compare different variables between groups and the variable importance in projection (VIP) values were calculated and used to select important features (VIP cut-off value of 1.0).

## Results and discussion

### Plant extraction

*T*. *nilotica* belongs to the family *Tamaricaceae* and the genus *Tamarix*, which is composed of 50–60 species of flowering plants that grow in maritime or sandy environments [38]. Fresh shoots of plant were collected, dried, and extracted as described in the materials and methods section.

Microwave-assisted extraction is relatively a new technique for easy and rapid extraction of phytoconstituents compared to conventional extraction methods [39, 40]. One main advantage of this technique is the efficiency of using water as extraction solvent. Water has higher dielectric constant but lower dielectric loss than ethanol or methanol at ambient temperature and 2.45 GHz, which makes the rate of microwave energy absorption by water higher than the dispersion of energy [41]. The crude extraction and fractionation yields are presented in Table 1. The extraction yields (%) of TN50E and TNW were 33.8 ± 0.88 and 29.6 ± 2.38, respectively. These yields were relatively high and consistent with previously reported values for the same plant [42].

**Table 1:** Yields of total TN extracts and methanol fractions (% w/w), total phenolic content, and antioxidant activity.

Sample	Sample type	Yield (%)	Total phenolic content (mg GAE/g dry extract)	DPPH scavenging IC_50_ (μg/mL)
TN50E	Crude Extract	33.80 ± 0.88	200.27	35.85
TNW	Crude extract	29.60 ± 2.38	88.60	94.95
TNW10M	10% methanol fraction	15.20 ± 2.55	140.46	3.33
TNW20M	20% methanol fraction	2.98 ± 0.08	335.57	5.39
TNW30M	30% methanol fraction	2.46 ± 0.24	372.18	6.96
TNW40M	40% methanol fraction	1.44 ± 0.16	383.10	6.61
TNW50M	50% methanol fraction	0.82 ± 0.15	336.93	6.72
TNW60M	60% methanol fraction	0.56 ± 0.04	260.53	7.38
TNW70M	70% methanol fraction	0.13 ± 0.01	147.13	4.80
TNW80M	80% methanol fraction	0.08 ± 0.01	176.69	6.78
TNW100M	100% methanol fraction	0.04 ± 0.00	nd*	nd

Abbreviations: TN50E (*T*. *nilotica* 50% ethanol extracts), TNW (*T*. *nilotica* aqueous extract), TNW10 –TNW100M (*T*. *nilotica* aqueous extract 10% to 100% methanol fractions). Yields are expressed as weight % of original material (mean ± standard deviation; n = 3). nd: not determined.

Methanol fractions showed comparable DPPH scavenging activity (IC_50_ ≤ 8 μg/mL) to ascorbic acid (IC_50_ = 5.6 μg/mL), and higher antioxidant activity when compared to crude aqueous and ethanol extracts (IC_50_ = 94.95 and 35.85 μg/mL, respectively). The strong antioxidant activity of plant extracts is often correlated with a higher phenolic content [43]. In general, the total phenolic content ranged from 88.60 to 383.10 mg GAE/g. The radical scavenging activity and total phenolic content of aqueous methanolic extracts and sub-extracts of *T*. *nilotica* flowers have been previously estimated and ethyl acetate extract has shown the highest antioxidant activity (IC_50_ = 7.25 ± 0.86 μg/mL) [42]. *In vivo* antioxidant activity of *T*. *nilotica* flowers extracted by maceration in 80% aqueous ethanol has also been determined by measuring tissue glutathione levels in alloxan-induced diabetic rats [44]. An increase in glutathione levels to near healthy levels was obtained after treatment with the plant extracts.

### α-glucosidase inhibition

**Fig 1:**
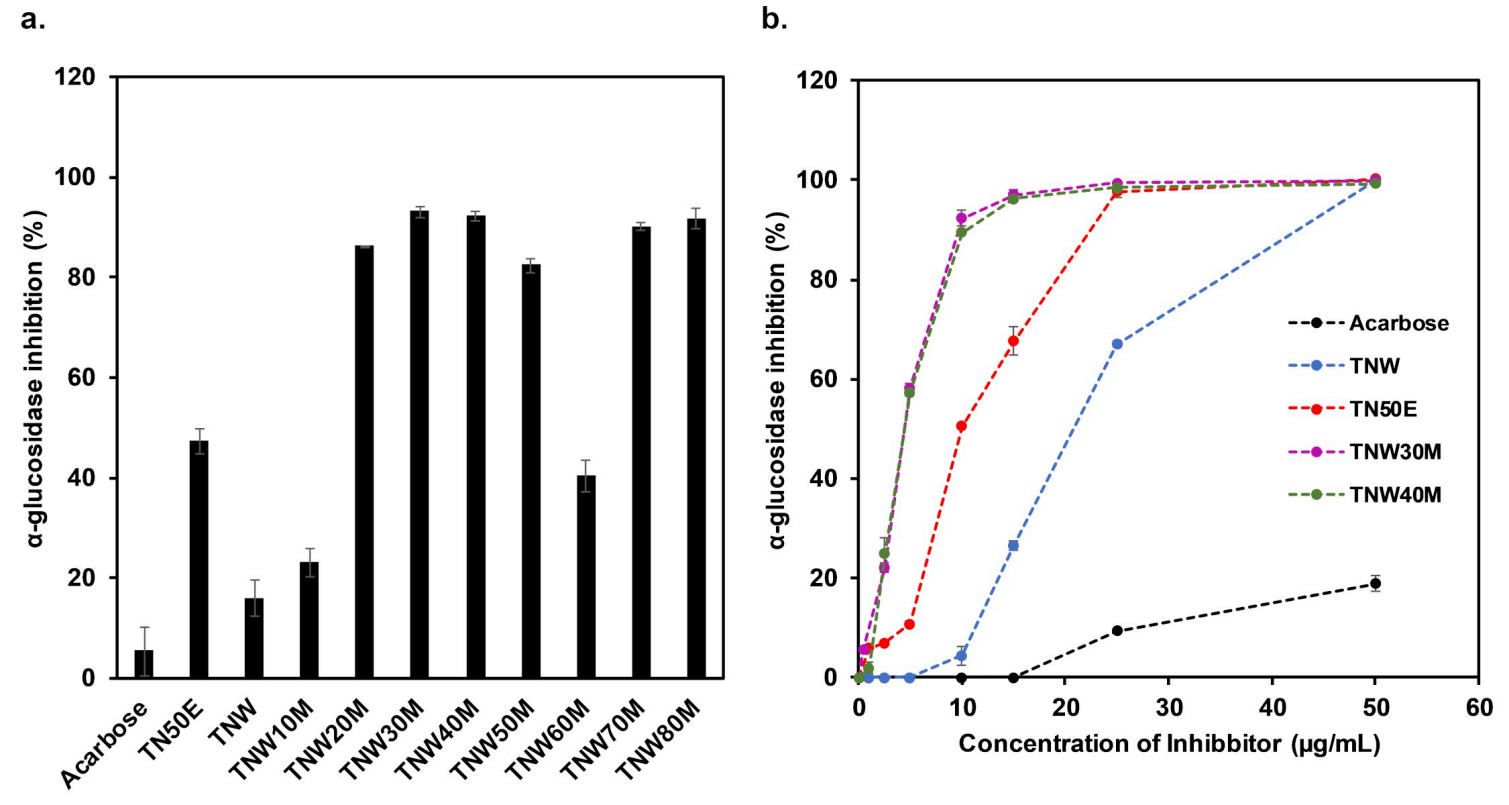
α-Glucosidase inhibitory activity of TN extracts. **(a)** α-Glucosidase inhibitory effect of different TN extracts compared to acarbose. A concentration of 10 μg/mL of TN extracts or acarbose was used in this assay. Results are expressed as percentage inhibition relative to the control without inhibitor. Mean values are presented and bars represent standard deviations (n = 3). **(b)** Concentration-dependent inhibition of TNW, TN50E, TNW30M, and TNW40M compared to acarbose.

TN50E, TNW, and all the methanol fractions prepared from the TNW extract were evaluated for their α-glucosidase inhibitory effect compared to the positive control acarbose (10 μg/mL final concentration). Both the crude TNW and TNE extracts demonstrated a significantly higher α-glucosidase inhibitory effect than pure acarbose (Fig 1A). Fractionation of TNW extract with 10% - 80% methanol resulted in increased inhibitory activity overall (23% - 93% inhibition), with the highest observed inhibition in the TNW30M, TNW40M, TNW70M, and TNW80M fractions. Interestingly, fractions TNW30M and TNW40M showed to have the highest phenolic contents (372.18 mg GAE/g and 383.10 mg GAE/g, respectively) as well suggesting a role for these molecules in the inhibitory activity (Table 1). A similar association between the measured phenolic contents, the antioxidant and free radical scavenging activities, and the inhibitory effects against α-amylase and α-glucosidase was previously observed in *Elateriospermum tapos* shell extracts and *Hyophorbe lagenicaulis* leaf extracts [45, 46]. The TNW10M and TNW60M fractions displayed significantly lower inhibitory action when compared to the other methanol fractions. Based on these results, it is evident that the fractionation with 30, 40, 70, and 80% methanol resulted in significantly enhanced activity against α-glucosidase. This can be due to the interference of compounds in the crude extract and other fractions with the measured activity, and/or the isolation of higher concentrations of the inhibitory molecule(s) following fractionation with these concentrations of methanol.

Inhibition by TN extracts occurred in a concentration-dependent manner (Fig 1B), and the concentration resulting in 50% α-glucosidase inhibition was determined for the TNW and TN50E crude extracts as well as for the TNW30M and TNW40M fractions. Results showed the highest inhibitory effect with TNW30M and TNW40M (IC_50_ = 5.2 μg/mL) followed by TN50E (IC_50_ = 12.5 μg/mL) (Table 2). IC_50_ values were up to 30-folds lower for TN extracts compared to acarbose (IC_50_ = 151.1 μg/mL) and lower than previously reported values using MeOH stem extracts from *T*. *aphylla* (IC_50_ = 8.41 μg/mL) in the case of TNW30M and TNW40M [30]. In addition, TN extracts showed lower IC_50_ values compared to other recently described α-glucosidase-inhibiting plant extracts including root bark extract of *Aralia taibaiensis* (IC_50_ = 410 μg/mL), ethyl acetate extracts of *Radix Astragali* (IC_50_ = 14–1400 μg/mL), aqueous leaves extracts of *Coccinia grandis* (IC_50_ = 77.66 μg/mL), and *n*-hexane extracts of *Citrullus lanatus* or watermelon (IC_50_ = 34.4 μg/mL) [47–50]. Taken together, it is clear that there may be strongly active α-glucosidase inhibitors in TN extracts. It is also remarkable that these crude extracts were able to inhibit α-glucosidase at much lower concentrations compared to pure acarbose. Further purification of the active fractions could potentially result in compounds with potential IC_50_ values in the ng/mL range. Indeed, fractionation of the crude TNW extract resulted in fractions with 5 times higher inhibition activity (TNW crude IC_50_ = 25 μg/mL compared to TNW30M fraction IC_50_ = 5 μg/mL).

**Table 2:** α-Glucosidase inhibitory effect (IC_50_) of different TN extracts compared to acarbose.

Sample	IC_50_ (μg/mL)
Acarbose	151.14 ± 0.77
TN50E crude extract	12.56 ± 0.06
TNW crude extract	24.79 ± 0.33
TNW30M fraction	5.21 ± 0.02
TNW40M fraction	5.23 ± 0.005

Values are expressed as mean ± standard deviation.

### Kinetics of α-glucosidase inhibition by TN extracts

**Fig 2:**
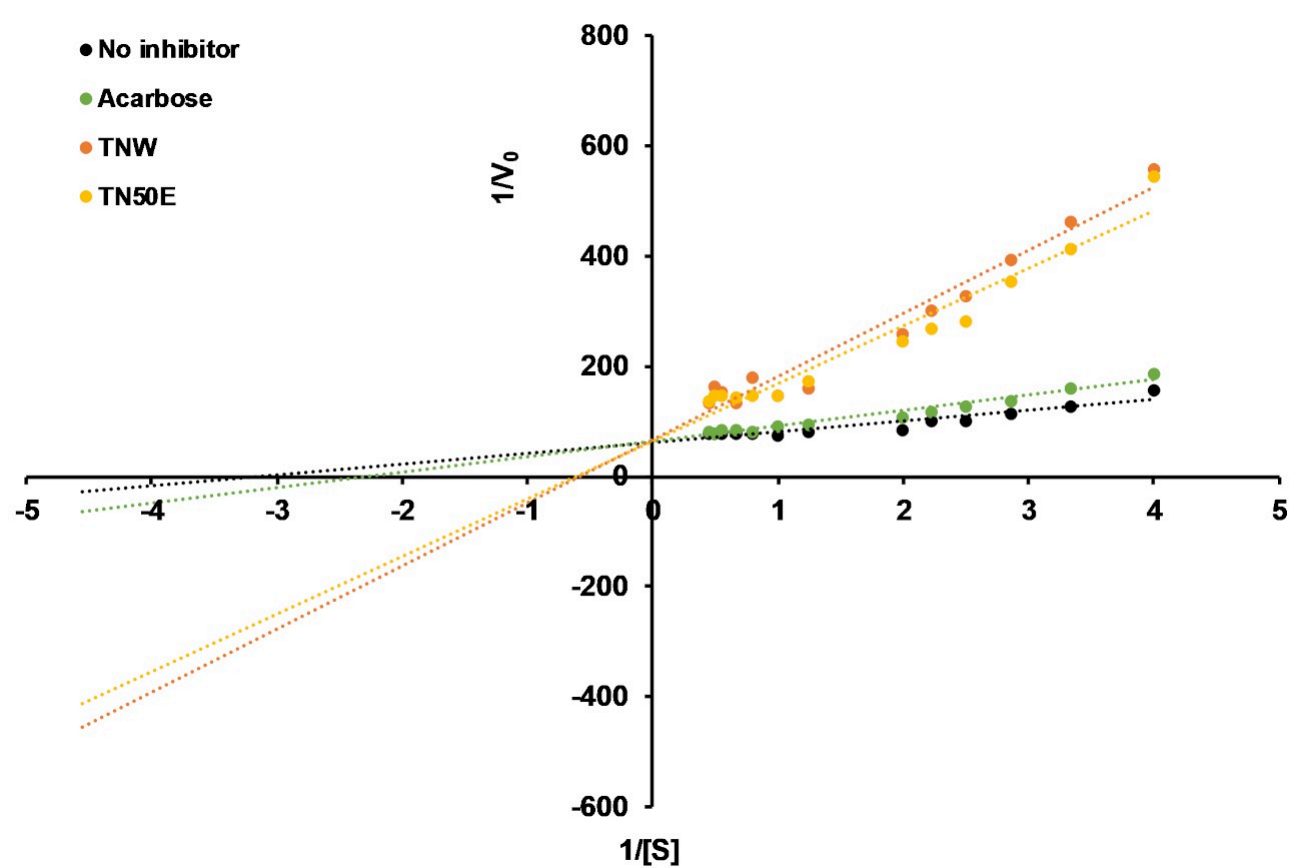
Lineweaver-Burk plot of TN extracts inhibitory activity. Lineweaver-Burk plot in the absence (black) and presence of IC_50_ (μg/mL) equivalent of Acarbose (green), TNW (orange) and TN50E extract (red). V_0_ represents the initial rate of the reaction and [S] the concentration of pNPG in the reaction.

The inhibition type and kinetic parameters of α-glucosidase in the presence of TNW and TN50E extracts were identified from the Linewaver–Burk and Michaelis-Menten plots and compared to acarbose and the uninhibited control (Fig 2).

No changes in *V*_*max*_ were observed for both TN extracts while the *K*_*m*_ values increased 5 and 4-folds for TNW (*K*_*m*_ = 1.5 mM) and TN50E (*K*_*m*_ = 1.3 mM), respectively, compared to the uninhibited control indicating a competitive inhibition mechanism (Table 3). The *K*_*m*_ values in the cases of both TN extracts were also significantly higher than the *K*_*m*_ value in the presence of acarbose (*K*_*m*_ = 0.4 mM) showing again a higher α-glucosidase inhibitory effect of the plant material. This was also reflected by the low calculated *K*_*i*_ values for the TN extracts (*K*_*i* TNW_ = 3.2 μg/mL; *K*_*i* TN50E_ = 1.7 μg/mL) compared to acarbose (*K*_*i*_ = 103.5 μg/mL).

**Table 3:** Kinetic parameters of α-glucosidase inhibition in absence and presence of *T*. *nilotica* extracts (TNW or TN50E) compared to acarbose.

Sample	*K*_*m*_ (mM)	*V*_*max*_ (μmole/min)	*K*_*i*_ (μg/mL)	Variation of *K*_*m*_ and *V*_*max*_	Type of Inhibition
No inhibitor	0.310 ± 0.032	0.016 ± 0.001			
Acarbose	0.422 ± 0.036	0.016 ± 0.001	103.559 ± 6.290	↑ *K*_*m*_	Competitive
				*= V*_*max*_	
TN50E	1.285 ± 0.025	0.013 ± 0.001	1.734 ± 0.039	↑ *K*_*m*_	Competitive
				*= V*_*max*_	
TNW	1.545 ± 0.089	0.014 ± 0.001	3.206 ± 0.203	↑ *K*_*m*_	Competitive
				*= V*_*max*_	

Values are expressed as mean ± standard deviation.

Competitive inhibition by TN extracts suggests that the responsible molecule(s) is/are structurally recognized by the active site of α-glucosidase used in this study. Previously studied plant extracts exhibiting competitive α-glucosidase inhibition include ethanol leaf extracts of *Antidesma celebicum*, aqueous fraction of *Stenochlaena palustris* extract and ethyl acetate extracts of *Equisetum arvense* [51–53].

**Fig 3:**
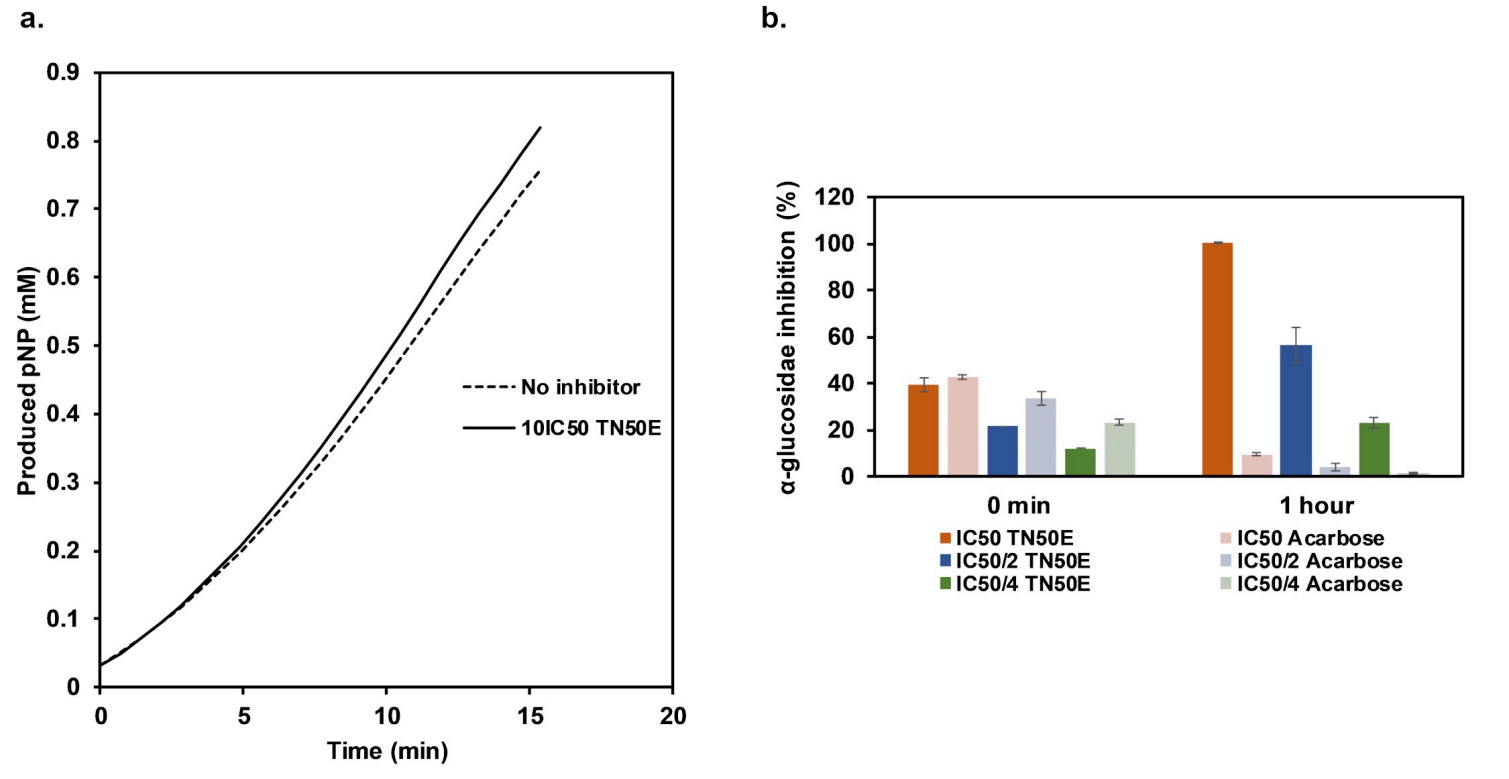
Reversibility of α-glucosidase inhibition and effect of pre-incubation. **(a)** Reversibility of α-glucosidase inhibition determined by rapid dilution assay. Produced pNP (mM) was determined after incubation of the enzyme in the absence (no inhibitor) or presence of TN50E extract. Initial rates were measured for 15 min after dilution of the enzyme-inhibitor complex with substrate to initiate the reaction. **(b)** α-glucosidase inhibition in the presence of IC_50_, IC_50_/2 and IC_50_/4 (μg/mL) of TN50E extract or acarbose before and after 1 h of pre-incubation with inhibitor. Mean values are presented and bars represent standard deviations.

To test for the reversibility of α-glucosidase inhibition by TN extracts, rapid dilution assays were performed. After rapid and large dilution of the enzyme-inhibitor complex, the production rate of pNP in the reaction with TN50E was comparable to the control without inhibitor (Fig 3A). This indicates a recovery of the enzymatic activity and a rapidly reversible inhibition by this extract that eliminates the suicide-inhibition hypothesis.

Commercially available antihyperglycemic drugs inhibiting intestinal α-glucosidase all act as competitive inhibitors [54]. Enzyme inhibition is a key mechanism in modern drug therapy and reversible, competitive inhibitors are highly favored since they have in general high specificity towards the targeted enzyme reducing undesirable effects caused by non-specific binding [55]. In addition, when inhibited by a competitive molecule the overall structure of the enzyme is not affected and the natural catalytic activity is retrieved upon treatment cessation.

To further investigate the inhibitory mechanism of α-glucosidase by TN50E extract, the enzyme was pre-incubated with different concentrations of extract for 1 h before the onset of the reaction. A remarkable increase in α-glucosidase inhibition was detected with all tested TN50E concentrations (Fig 3B), and a 2.58-folds shift in the determined IC_50_ was observed after pre-incubation. This mechanism of action has been widely described for CYP450 inhibitory drugs and was explained by the potential formation of inhibitory metabolites or by mechanism-based inhibition [56]. The reversibility of the reaction with TN50E extract leads to the assumption that the time-dependent inhibition is a result of the reversible interaction of the enzyme with metabolite(s) generated *in situ*. Another possible explanation is the reversible slow-binding of the inhibitor to the enzyme’s active site. This mode of inhibition was recently observed with Artoindonesianin W, a flavone isolated from bark extracts of *Artocarpus elasticus* [57].

### Thermal and pH stabilities

**Fig 4:**
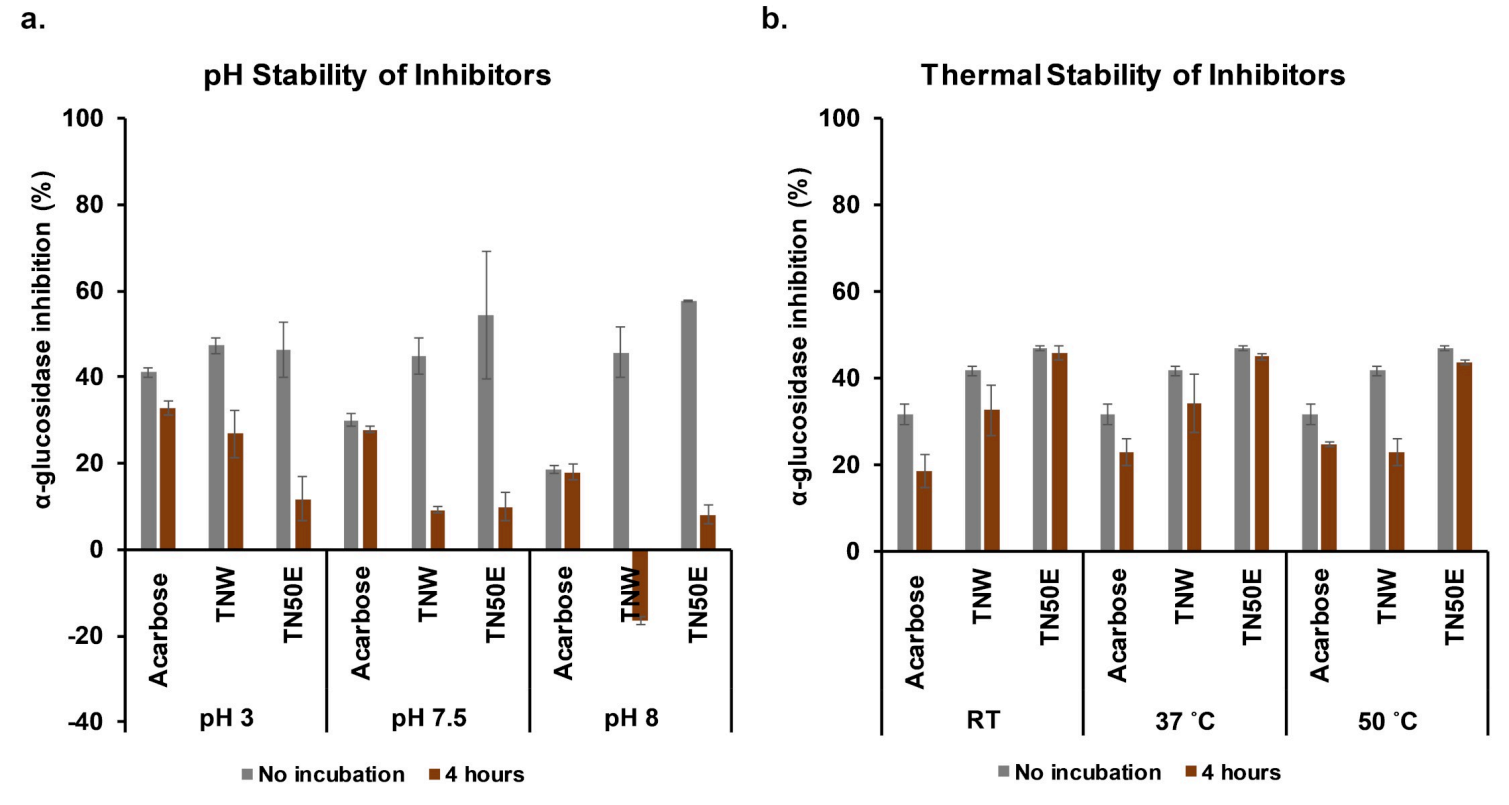
Thermal and pH stability of TN extracts. **(a)** pH stability of TN extracts (TNW and TN50E) compared to acarbose. Extracts were incubated at pH 3, 7.5 and 8 for 4 h at 37°C and their inhibitory effect was determined in the standard assay (pH 6.8). **(b)** Thermal stability of TN extracts (TNW and TN50E) compared to acarbose. Extracts were incubated at room temperature (RT), 37°C and 50°C for 4 h their inhibitory effect was determined in the standard assay (37°C). Mean values are presented and bars represent standard deviations.

To evaluate the stability profiles of TNW and TN50E extracts in the digestive organs, the plant materials were pre-incubated at different pH conditions mimicking the average pH conditions of the digestive tract [58]. Their α-glucosidase inhibitory effect was then assessed following the standard assay (pH 6.8) and compared to the control (TNW and TN50E without incubation) and acarbose. The inhibitory activity decreased significantly compared to the control for both TN extracts in the pH range 3–8 (Fig 4A). This effect was observed after prolonged (4 h) incubation and was strongest at pH 7.5 and 8. Unexpectedly, TNW pre-incubated at pH 8 showed an enhancing effect on α-glucosidase activity. The activation effect under this condition can be explained by either the chemical transformation of the metabolite(s) responsible for the inhibitory effect at pH 8 or the interaction between the enzyme and generated metabolites resulting in the protection of its active site against inhibition. Aqueous alkali condition for example is associated with increased hydrophilicity in carboxylic acid-containing molecules and flavonoids containing free phenolic groups [59]. On the other hand, acarbose was stable after 4 h in all tested pH conditions compared to the control without incubation but showed an overall decrease in activity at pH higher than 7.5.

The thermal stability of TNW and TN50E extracts was also investigated. Three temperatures were tested simulating storage (RT i.e. 25°C), human body (37°C), and extreme (50°C) conditions. The plant extracts were incubated for 4 h before performing the standard α-glucosidase inhibition assay (37°C). Results were compared to the control (without incubation) and acarbose. TN50E showed a high stability profile at all tested temperatures (Fig 4B). TNW on the other hand lost 50% of its activity after 4 h at 50°C. The observed results suggest a difference in the composition of the two extracts with the molecules extracted with 50% EtOH being more resistant to thermal variations and therefore promising candidates for drug development. A similar finding was recently reported demonstrating that ethanol has a stabilizing effect on the abundance and resistance of phytochemicals to high temperatures [33]. The thermal stability of a drug is of high pharmaceutical importance as it is directly related to the pharmacological activity and the shelf-life of the product.

### UHPLC Q-TOF MS analysis

**Fig 5:**
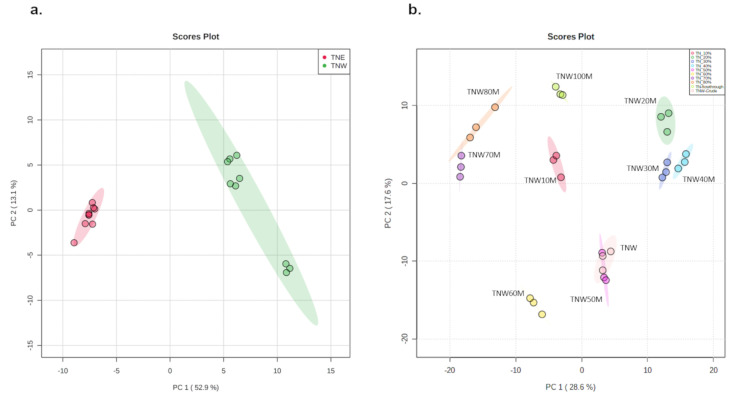
Multivariate analysis of phytochemicals identified in TNW, TN50E extracts and methanolic fractions. (**a**) Score plot of PCA analysis comparing TNW (green) and TN50E (red) extracts. (**b**) Score plot of PCA analysis comparing TNW methanolic fractions. MetaboAnalyst software was used to perform the analysis.

Previous studies have described a broad range of compounds produced by *T*. *nilotica* including carbohydrates, phenols, flavonoids, terpenoids, steroids, tannins and cardiac glycosides [38]. In this study, the phytoconstituents of TNW and TN50E extracts and the methanolic fractions were analyzed by UHPLC Q-TOF MS and compared to highlight metabolites potentially responsible for the inhibitory activity. A total of 577 known and 2032 unknown compounds were identified and their relative abundance was determined. The extracts were found to be in general rich in lipids, flavonoids, terpenes and carboxylic acids; however, the abundance and distribution of these molecules varied significantly between TNW and TN50E extracts, and TNW and the methanolic fractions. The two-dimensional principal component analysis (PCA) score plot showed that PC1 and PC2 were responsible for 52.9% and 13.1% of the variation, respectively, indicating a strong distinction between the chemical constituents in TNW and TN50E samples (Fig 5A).

A clear separation into six distinct clusters was also observed from the PCA plot of the methanolic fractions (Fig 5b). The variations between these fractions were less pronounced in some cases with specific fractions clustering together. Interestingly, fractions forming clusters (TNW20M, TNW30M and TNW40M; TNW70M and TNW80M) exhibited almost identical inhibitory activity except for TNW and TNW50M extracts. The detected activity can therefore be linked to the chemical composition and the presence of inhibitor(s) in these fractions.

**Fig 6:**
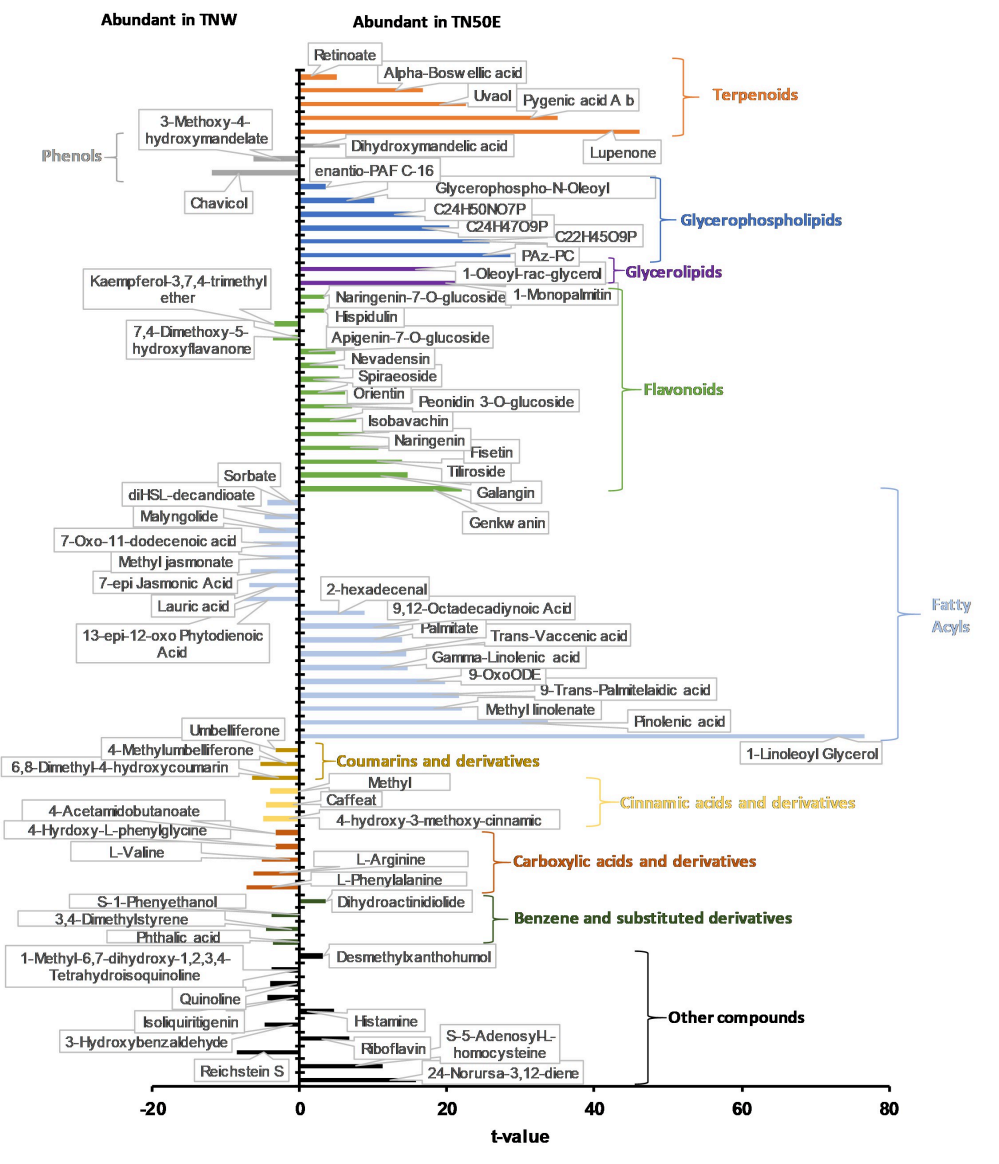
Molecules contributing to the difference between TNW and TN50E. Identified phytoconstituents whose relative abundance are significantly different between TNW and TN50E extracts (*p* value < 0.01). The chemical classifications and sub-classifications are presented for each metabolite.

Molecules contributing to considerable variations between TNW and TN50E extracts were identified by t-tests (*p* values < 0.01). The results highlighted 74 compounds that displayed significant variation in abundance between extracts. The molecules and their chemical classification and distribution between the two samples are shown in Fig 6. Terpenoids, glycerophospholipids, glycerolipids, flavonoids, and fatty acyls were in general significantly more abundant in TN50E extract, while TNW was rich in coumarins, cinnamic acid, carboxylic acids and benzene derivatives.

The large distance between the two clusters grouping TNW20M-TNW40M and TNW70M/TNW80M (Fig 5b) also suggests that the molecule(s) responsible for the α-glucosidase inhibitory activity are different in each cluster. To examine these hypotheses, the compounds contributing to the observed variation between the two most active and two least active cluster groups were identified by variable importance in projection (VIP) scores. The analysis identified 101 compounds whose abundance was significantly higher in the active fractions compared to the less active fractions TNW10M/TNW60M (Table 4). Among these molecules, 61 and 52 compounds were significantly more abundant in the groups TNW30M/TNW40M and TNW70M/TNW80M, respectively compared to the least active group TNW10M/TNW60M. Twelve molecules were common between the active extracts and these compounds were significantly less abundant in TNW10M and TNW60M. However, no previous studies investigating the role of the common molecules as α-glucosidase inhibitors exist. Based on the work presented here, future studies aimed at determining if these compounds can alone or in combination inhibit α-glucosidase are warranted.

**Table 4:** Compounds with significantly higher abundance in TNW30M/TNW40M and TNW70M/TNW80M compared to TNW10M/TNW60M fractions.

		TNW10M	TNW30M	TNW40M	TNW60M	TNW70M	TNW80M
Chemical Classification	Compound	Relative Abundance (*10^2^)					
**Terpenoids**	Sclareol	0.00	507.36	271.14	2.40	1.35	0.79
	Simulanoquinoline	0.00	105.02	52.51	0.00	0.00	0.00
	Lactapiperanol C	0.00	58.60	35.19	0.71	0.81	0.00
	gamma-Eudesmol rhamnoside	0.21	0.60	0.75	1.75	121.20	198.50
	Petasitin	0.00	0.00	0.00	49.96	280.20	82.66
	L-Menthyl acetoacetate	2.27	0.23	0.18	0.93	12.80	25.52
	Ginkgolide A	0.00	0.20	0.22	1.36	7.26	9.35
**Lipids**	Sorbate	11.70	1823.65	945.61	119.24	11.25	3.28
	Undecanedioic acid	13.42	148.70	74.35	4.32	1.23	2.40
	Quassinol	0.00	142.36	85.33	0.00	0.00	0.00
	12,13-DiHODE	1.66	6.06	110.46	0.00	1.74	1.40
	(-)-11-Hydroxy-9,15,16-trioxooctadecanoic acid	0.10	15.93	100.28	7.70	6.23	0.00
	(±)9-HpODE	0.00	63.95	31.97	3.30	0.00	0.00
	13,14-Dihydro PGF-1a	4.79	27.71	72.33	0.00	0.00	0.00
	Isobutyl 10-undecenoate*	0.17	29.97	14.99	5.92	36.24	13.81
	Tsangane L 3-glucoside*	0.00	43.90	21.95	2.49	5.82	0.98
	Tetrahydrocortisol*	0.00	8.58	33.93	1.55	14.50	15.12
	3-Hydroxy-6,8-dimethoxy-7(11)-eremophilen-12,8-olide	0.05	25.79	21.56	1.67	5.55	6.32
	(±)-(E)-3-Methyl-4-decen-1-yl acetate	0.00	21.43	42.17	0.00	0.00	0.00
	(R)-3-Hydroxy-tetradecanoic acid	1.18	11.29	14.12	9.02	2.74	1.48
	Geranyl 2-methylbutyrate*	0.00	3.11	2.56	0.98	15.40	2.41
	Phenethyl decanoate	7.20	0.00	0.00	16.78	250.02	110.60
	Lauroyl diethanolamide	2.27	0.00	0.00	6.23	142.90	53.06
	Estradiol-17Alpha	0.29	0.71	0.70	6.48	26.21	27.09
	Linoleate	2.21	0.00	0.00	0.93	42.34	2.92
	Gamma-Linolenic acid	0.00	0.00	0.00	0.00	17.84	9.60
	(3R,7R)-1,3,7-Octanetriol	2.41	0.00	0.00	0.46	4.24	7.52
**Flavonoids**	Sexangularetin 3-rutinoside	0.00	62.15	559.29	0.00	2.73	3.97
	Pollenitin*	6.97	103.89	106.11	25.35	174.35	103.88
	Sissotrin	0.00	88.87	79.35	0.00	0.00	0.00
	3,3’,4’,5,6,8-Hexamethoxyflavone	0.00	11.03	7.59	0.00	0.00	0.41
	Glicoisoflavanone	0.00	1.68	1.98	0.29	83.09	60.27
	Isoartocarpesin	0.00	0.21	0.11	0.43	9.99	31.98
	Glyceollidin I	0.00	0.00	0.00	0.00	32.92	59.65
	Peonidin 3-O-glucoside	2.25	0.00	0.00	0.00	50.00	6.17
	Floribundoside	0.61	0.51	0.69	0.35	19.57	9.33
	Isoswertiajaponin	0.00	0.70	1.10	1.43	38.48	26.38
	Isoartocarpesin	0.00	0.21	0.11	0.43	9.99	31.98
	6’’-O-Acetylglycitin	0.00	8.86	4.43	0.00	15.06	24.87
	Dalbergioidin	0.00	0.00	0.00	0.92	47.88	25.32
**Carbohydrates and carbohydrate conjugates**	a-L-Fucopyranosyl-(1->2)-b-D-galactopyranosyl-(1->2)-D-xylose	0.00	342.87	225.20	26.76	0.00	2.06
	2,3-Butanediol glucoside	0.00	104.24	98.73	0.41	1.84	6.02
	Pteroside D	0.85	86.85	43.43	0.00	0.00	0.00
	Glucocaffeic acid	0.00	0.56	2.09	88.82	2094.39	3739.80
	Gluconic acid	0.00	0.00	0.00	21.74	267.31	168.94
	Garcimangosone D	0.00	0.00	0.00	4.04	41.70	104.88
**Benzene and substituted derivatives**	2-Methylbenzoic acid	12.70	1440.27	756.24	0.00	0.00	0.00
	1-Methyl-2-propylbenzene	0.00	13.91	1357.90	3.52	0.00	0.44
	Methyl phenylacetate	0.00	40.84	1090.29	2.62	0.97	2.28
	Styrene*	21.33	38.34	78.38	17.69	74.09	17.03
	2-(3,4-dimethoxyphenyl)ethanamine	0.00	43.09	28.15	1.28	0.00	0.00
	3,4-Dihydroxybenzoate	0.96	16.09	13.98	0.00	0.00	0.92
	4R,5R,6S-Trihydroxy-2-hydroxymethyl-2-cyclohexen-1-one 6-(2-hydroxy-6-methylbenzoate)	0.00	7.69	9.81	0.00	0.00	0.00
	Prenyl benzoate	1.37	6.30	24.16	714.69	5851.95	10429.69
	Hamamelitannin	0.00	0.56	1.85	6.87	359.99	350.17
	Homoveratric acid	0.00	0.00	0.77	0.00	297.39	96.91
**Phenols and cinnamic/coumaric acids derivatives**	1-(3-Hydroxy-4-methoxyphenyl)-1,2-ethanediol	0.00	754.41	524.24	9.82	1.14	1.12
	2-Benzylidene-1-heptanol	0.00	60.06	347.15	11.86	0.00	0.00
	N-trans-Feruloyl-4-O-methyldopamine*	0.00	169.24	84.62	12.35	0.00	0.00
	3,4,5-Trimethoxycinnamic acid*	0.00	7.16	159.31	0.00	6.05	0.64
	3-(4-Methoxyphenyl)-2-propenal	2.94	71.20	60.24	26.23	4.64	1.93
	2,6-Dimethoxy-4-propylphenol	4.93	8.55	17.96	0.00	3.15	1.03
	Gravolenic acid	1.71	2.26	3.23	4.00	4221.63	1541.46
	p-Hydroxyphenethyl trans-ferulate	0.00	0.25	0.60	0.00	133.38	285.23
	p-Coumaric acid	9.64	10.52	8.39	10.01	36.68	25.98
	Feruloyl-2-hydroxyputrescine	0.00	0.00	0.00	0.00	52.58	18.69
	Methyl cinnamate	49.07	2.21	1.97	1.95	35.50	9.51
**Carboxylic acids**	Vinylacetylglycine	56.06	0.00	0.00	72.64	1897.28	5397.06
	Pipecolate	50.18	33.49	35.80	66.16	1735.07	1175.00
	Genipinic acid	4.50	17.51	19.87	18.07	70.52	48.13
	Glutaric acid	2.22	0.00	0.00	0.00	17.08	3.48
**Branched unsaturated hydrocarbons**	Santene	0.00	754.41	524.24	9.82	1.14	1.12
	(6E,8E)-4,6,8-Megastigmatriene*	0.10	20.64	21.77	8.61	10.82	31.60
**Benzodioxoles**	N-(Heptan-4-yl)benzo[d][1,3]dioxole-5-carboxamide	0.00	2243.33	1121.67	0.00	0.00	0.00
	Sesamolin	0.00	785.37	463.03	11.99	10.41	0.00
**Carbonyl compounds**	(Z)-Tamarindienal	9.08	72.02	826.48	5.01	6.78	1.02
	2,6,6-Trimethyl-1-cyclohexen-1-acetaldehyde	3.03	69.84	121.05	0.00	0.00	0.00
	2-Methylacetophenone	0.00	110.64	74.73	0.00	0.00	0.00
**Chromones**	Mycochromone	0.00	1535.75	1724.54	5.17	0.00	0.00
**Purine nucleotides**	2’-Deoxyguanosine 5’-monophosphate	0.00	1199.91	866.15	0.00	0.00	0.00
**Alkanolamine**	2-Amino-2-methyl-1-propanol	0.00	502.46	377.51	4.44	0.00	0.00
**Hydroxybenzaldehyde**	3-Hydroxybenzaldehyde	7.01	341.47	170.73	8.64	0.00	0.00
**Naphthopyrans**	Momilactone B	0.00	312.43	156.22	1.37	0.00	0.00
**Anthracenes**	Rhein	0.00	230.87	181.30	0.00	1.16	1.70
**Alkaloids and derivatives**	Norisodomesticine	0.00	178.60	259.47	63.55	0.00	0.00
**Kavalactones**	11-Hydroxyyangonin	0.00	68.71	238.53	0.00	0.44	0.00
**Heteroaromatic compound**	(E)-6-Methyl-6-(5-methyl-2-furanyl)-3-hepten-2-one	9.24	98.93	184.76	27.93	12.95	6.80
**Diarylheptanoids**	Cyclocurcumin*	1.84	43.03	22.97	22.76	22.24	0.00
**Azepines**	2,3,6,7-Tetrahydrocyclopent[b]azepin-8(1H)-one	0.00	51.43	29.21	2.16	3.12	0.00
**Trioxane**	2,4,6-Triethyl-1,3,5-trioxane	0.00	27.88	23.15	0.00	2.17	0.94
**Amino acids and derivatives**	2-Aminomuconic acid semialdehyde	3.20	58.02	58.65	0.00	2.83	0.00
	Melilotocarpan A	0.00	25.00	21.32	0.00	0.00	1.37
**Hydroxy acids and derivatives**	(S)-Malate	48.84	13.15	27.25	3.12	1.73	1.38
**Diol**	Trans-cyclohexane-1,2-diol*	0.00	0.00	24.47	0.00	7.56	4.26
**Amines**	1-Phenylethylamine*	0.17	8.17	4.08	3.67	13.38	0.00
**Porphyrins**	Harderoporphyrin	0.00	0.00	0.00	0.00	501.03	132.34
**Tetrahydroisoquinolines**	Hydrocotarnine	2.78	3.51	3.54	12.97	109.68	277.46
**Xanthone**	Garbogiol	0.00	0.00	0.13	0.00	71.88	91.56
**Furanoid**	Lirioresinol A	0.24	0.00	0.07	1.67	50.51	94.12
**Pyranones and derivatives**	Kojic acid	0.00	1.69	2.68	0.00	48.03	39.20
**Benzopyrans**	(5alpha,8beta,9beta)-5,9-Epoxy-3,6-megastigmadien-8-ol	15.33	11.32	10.31	18.74	16.84	55.18

All the presented metabolites showed VIP score ≥ 1 in the contribution to the variability between the most active fractions TNW30M/TNW40M (highlighted in orange) or TNW70M/TNW80M (highlighted in blue) and the least active fractions TNW10M/TNW60M. Common molecules that showed high abundance in TNW30M/TNW40M and TNW70M/TNW80M are indicated by a star. Compounds are grouped according to their chemical classification.

A total of 5 terpenoids, including 4 triterpenoids, and 1 retinoid were identified and found to be significantly more abundant in TN50E extract (Fig 6). Terpenoids are hydrophobic molecules and dissolve very poorly in water and readily in organic solvents, which can explain their abundance in TN50E extract [60]. The α-glucosidase inhibitory activity of terpenoids has been previously described [61, 62]. In *Pelliciera rhizophorae* extracts, pentacyclic triterpenoids were the most potent α-glucosidase inhibitors acting in a competitive reversible manner and binding to the active site of the enzyme through hydrophobic interactions [63]. Among the identified triterpenes in TN extracts, two pentacyclic triterpenoids: pygenic acid A and lupenone were exclusively detected in TN50E extract. These compounds have been previously shown to exhibit potent activity against α-glucosidase enzyme with IC_50_ values of 30.18 μg/mL and 112.36±0.18 μM for pygenic acid A and lupenone, respectively [64, 65]. The highly active methanolic fractions also contained terpenoids that were significantly more abundant in these fractions compared to the least active fractions TNW10M/TNW60M (Table 4). Two diterpenoids (lactapiperanol C and sclareol) were particularly more abundant in TNW30M/TNW40M, while TNW70M/TNW80M showed higher sesquiterpenoids content. Sesquiterpenoids have also been described as α-glucosidase inhibitors. One new sesquiterpene, has been recently isolated from the methanolic extracts of *Teucrium mascatense* and exhibited strong inhibitory activity against α-glucosidase with an IC_50_ value of 121.2 μM as compared to acarbose (IC_50_  =  942.0 μM) [66]. Docking studies revealed that this molecule binds strongly to the catalytic site of the enzyme.

Extraction with 50% ethanol also contributed to increased detection of flavonoids with only 2 compounds (O-methylated flavonoids) out of 15 being more abundant in the aqueous extract. The highly active fractions TNW70M and TNW80M were particularly rich in flavonoids including flavonoid glycosides, isoflavonoids, flavans and flavones. Flavonoids are widely distributed in the plant kingdom and are known for their antioxidant, anti-inflammatory and anti-carcinogenic properties with many molecules already available in the market as natural supplements [67]. Their role as α-glucosidase and α-amylase inhibitors have also been described and the inhibitory mechanisms of different subclasses of flavonoids were studied [68]. Molecular docking analysis identified structural features that determine the α-glucosidase inhibitory potentials of flavonoids. The presence of 3-OH group in the C-ring, 4’-OH and 3’,4’-(OH)_2_ in the B-ring of flavones have been associated with higher inhibitory activity while bulkier molecules with -O-methyl showed a weaker effect [68]. Further studies on flavonoids glycosides and O-methylated flavonoids from *T*. *gallica* showed that the presence of glucuronic acid and its methyl ester at the C-3 position enhanced the inhibitory activity [32]. In addition, recent molecular docking results revealed that the presence of functional groups that increase the hydrophobicity of the compound enhanced the hydrophobic interactions with F157 and R312 residues of the enzyme, stabilizing the binding of the inhibitor and contributing to the inhibitory activity [57]. The detected flavonoids in the TN extracts previously known to exhibit high inhibitory activity included flavones (galangin, fisetin), flavans, and flavonoids glycosides (tiliroside, peonidin 3-O-glucoside, orientin) with structural features favoring α-glucosidase inhibition suggesting the implication of these molecules in the observed activity [69–72]. Peonidin 3-O-glucoside was detected in TN50E, TNW70M and TNW80M conditions and has been previously described to be as efficient (IC_50_ = 192.3 μg/mL) as acarbose (IC_50_ = 180.2 μg/mL) in inhibiting rat intestinal α-glucosidase [73]. Dalbergioidin, an isoflavanone that was abundant in TNW70M and TNW80M fractions has been found to efficiently inhibit α-glucosidase (IC_50_ = 412.6 μM) compared to acarbose (IC_50_ = 671.4 μM) [74]. The xanthone garbogiol has also been found to exhibit considerable α-glucosidase inhibition (IC_50_ = 21.2 μM) compared to acarbose (IC_50_ = 214.5 μM) [75].

Another class of molecules significantly contributing to the differences observed between samples is lipids. Identified molecules were glycerophospholipids, glycerolipids and fatty acyls (mainly fatty acids and linoleic acid derivatives). Lipids are structural components of plant cell membranes and they play important role in signaling and energy storage. In biotechnology, these molecules are valued for their nutritional and industrial applications [76]. Fatty acids have been also described to have antibacterial, anti-inflammatory and cardioprotective properties [77, 78]. The role of these molecules as α-glucosidase inhibitors has been researched in a few studies. Metabolite analysis of *Piper betle* (L.) ethanolic leaf extracts has associated the detected strong α-glucosidase inhibitory activity with the presence of palmitic, stearic, oleic, linolenic and linoleic acids [79]. Kinetic assays on oleic acid and linoleic acid have shown that the molecules bind to α-glucosidase forming a complex and inhibiting the enzyme competitively [80]. A stronger inhibitory effect has been observed with unsaturated fatty acids compared to saturated fatty acids and methyl esters form of unsaturated fatty acids [81]. In the current study, most of the identified lipids were unsaturated fatty acids and they were more abundant in TN50E extract and active methanolic fractions which might have contributed to the higher activity of these samples. Two of these molecules; pinolenic acid and 1-linoleoyl glycerol were in the top 5 compounds with the highest VIP score in the comparison between TNW and TN50E extracts. In addition, gamma-linolenic acid was common between TN50E and TNW70M/TNW80M and was significantly abundant in these samples.

TNW70M/TNW80M fractions also contained p-hydroxyphenethyl trans-ferulate which has previously shown strong inhibitory activity on yeast α-glucosidase (IC_50_ = 19.24 μmol/L), comparable to the reference quercetin (IC_50_ = 15.61 μmol/L) [82]. Other abundant metabolites in TNW30M/TNW40M with previously described α-glucosidase inhibitory activity included rhein which has shown strong activity with IC_50_ value of 5.68 μM [83]. Momilactone B was also abundant in these fractions and this molecule has been found to exert greater α-glucosidase suppression (IC_50_ = 612.03 μg/mL) as compared to acarbose (IC_50_ = 2549 μg/mL) [84].

Overall, 50% ethanol extraction, and the fractionation of the aqueous extract with 20–40% and 70–80% methanol enhanced the presence of molecules with α-glucosidase inhibitory properties. The presence of organic solvents such as ethanol and methanol in this case along with water enabled the extraction of all compounds that were soluble in both water and organic solvents. The potent activity obtained with crude TNW and its fractions is very promising as aqueous extraction provides an inexpensive and environmentally friendly alternative to the use of toxic and flammable solvents. In addition to the potential individual role that each of the detected molecules can play in α-glucosidase inhibition, their simultaneous presence and increase in the most active samples can suggest a synergistic action between these compounds.

## Conclusion

This study supported the importance of *T*. *nilotica* as a medicinal plant for diabetes and a source of promising α-glucosidase inhibitors. Both, aqueous and ethanol shoot extracts of this plant showed higher *in vitro* efficacy against yeast α-glucosidase enzyme compared to the approved α-glucosidase inhibiting drug acarbose. Methanol fractions of the aqueous extract showed improved activity with increasing concentration of methanol except for TNW60N. Metabolite profiling and comparison of the different samples revealed very distinctive profiles and the presence of polar and/or water-soluble compounds previously associated with α-glucosidase inhibition as well as compounds that have not yet been explored for α-glucosidase inhibitory activity. Further isolation of the detected compounds and the investigation of their *in vitro* and *in vivo* inhibitory activity and synergistic action can lead to the discovery of new safe and efficient antidiabetic drugs.

## Supporting information

S1 File Raw data figures. (RAR)
